# Gestational Weight Gain Is Associated with the Expression of Genes Involved in Inflammation in Maternal Visceral Adipose Tissue and Offspring Anthropometric Measures

**DOI:** 10.3390/jcm12216766

**Published:** 2023-10-26

**Authors:** Renata Saucedo, María Isabel Peña-Cano, Mary Flor Díaz-Velázquez, Aldo Ferreira-Hermosillo, Juan Mario Solis-Paredes, Ignacio Camacho-Arroyo, Jorge Valencia-Ortega

**Affiliations:** 1 Unidad de Investigación Médica en Enfermedades Endocrinas, Hospital de Especialidades, Centro Médico Nacional Siglo XXI, Instituto Mexicano del Seguro Social, Mexico City 06720, Mexico; sgrenata@yahoo.com (R.S.); aldo.nagisa@gmail.com (A.F.-H.); 2 Hospital de Gineco Obstetricia 221, Instituto Mexicano del Seguro Social, Toluca 50000, Mexico; isabelpenacano@hotmail.com; 3 Hospital de Gineco Obstetricia 3, Centro Médico Nacional La Raza, Instituto Mexicano del Seguro Social, Mexico City 02990, Mexico; mary.diaz@imss.gob.mx; 4 Department of Reproductive and Perinatal Health Research, Instituto Nacional de Perinatología Isidro Espinosa de los Reyes, Mexico City 11000, Mexico; juan.solis@inper.gob.mx; 5 Unidad de Investigación en Reproducción Humana, Instituto Nacional de Perinatología-Facultad de Química, Universidad Nacional Autónoma de México, Mexico City 11000, Mexico; camachoarroyo@gmail.com

**Keywords:** gestational weight gain, visceral adipose tissue, cytokines, birthweight, inflammation

## Abstract

Background: Adequate gestational weight gain (GWG) is essential for maternal and fetal health. GWG may be a sign of higher visceral adipose tissue (VAT) accretion. A higher proportion of VAT is associated with an inflammatory process that may play a role in the fetal programming of obesity. This study aimed to (1) compare the expression of genes involved in inflammatory responses (TLR2, TLR4, NFκB, IKKβ, IL-1RA, IL-1β, IL-6, IL-10, TNF-α) in the VAT of pregnant women according to GWG and (2) explore whether VAT inflammation and GWG are related to offspring anthropometric measures. Material and methods: 50 women scheduled for cesarean section who delivered term infants were included in the study. We collected maternal omental VAT, and the expression of genes was examined with RT-qPCR. Results: Women with excessive and with adequate GWG had significantly higher expressions of most inflammatory genes than women with insufficient GWG. Neonates from mothers with excessive GWG had greater birth weight and chest circumference than those from mothers with insufficient GWG. GWG was positively correlated with fetal birth weight. Conclusions: The VAT expression of most genes associated with inflammatory pathways was higher in excessive and adequate GWG than in pregnant women with insufficient GWG. Moreover, GWG was found to be positively associated with newborn weight.

## 1. Introduction

Gestational weight gain (GWG) is a critical physiological process that supports adequate fetal growth and development. Insufficient or excessive GWG is associated with a higher risk of adverse pregnancy outcomes [1]. Insufficient GWG confers a higher risk for small-for-gestational-age infants and preterm birth [2]. In contrast, women with excessive GWG are at increased risk of adverse obstetric outcomes, including cesarean delivery, gestational diabetes mellitus, and preeclampsia [3]. Moreover, excessive GWG is associated with postpartum weight retention and pre-pregnancy adiposity in subsequent pregnancies [4]. The neonates also have adverse consequences, such as macrosomia and being large for their gestational age [5]. In addition, women with excessive GWG and their offspring have lifelong health disturbances, including obesity and a higher risk of type 2 diabetes and cardiovascular disease [6].

The Institute of Medicine (IOM) has recommended the ideal GWG to ensure the optimal health of the mother and the offspring [7]. The IOM proposes less GWG for women with overweight/obesity than for target-weight women. It has been estimated that only 28–32% of pregnant women have adequate GWG, almost 25% have insufficient weight gain, and half of the pregnancies gain in excess of the IOM recommendations [2]. In this context, women with overweight and women with obesity have the highest prevalence of excessive GWG, which is becoming increasingly prevalent among pregnancies worldwide [8,9].

GWG includes the fetus, placenta, uterus, amniotic fluid, maternal blood volume, breast tissue, and adipose tissue (AT). Maternal body fat accounts for about 30% of GWG and provides an energy source for the fetus and the mother [10]. Body fat distribution changes throughout pregnancy; there is a gradual decrease in subcutaneous fat accretion and an increase in visceral fat accumulation from early to late gestation [11]. Women with overweight and women with obesity have a higher proportion of visceral adipose tissue (VAT) than lean women, and this is associated with VAT inflammation, characterized by the increased release of pro-inflammatory signals (leptin, macrophage chemoattractant protein 1, IL-6, and IL-1β) and reduced production of anti-inflammatory molecules (adiponectin, IL-10) [12]. Adipose tissue inflammation has been related to the development of maternal metabolic disorders such as insulin resistance. Growing evidence supports that it also plays a relevant role in the fetal programming of obesity [13].

GWG may be a sign of higher VAT accretion during pregnancy [14]. Nevertheless, the relationship between GWG and VAT inflammation and offspring anthropometric measures at birth has not been tested. Therefore, the aim of this study was to (1) compare the expression of genes involved in inflammatory responses (TLR2, TLR4, NFκB, IKKβ, IL-1RA, IL1-β, IL-6, IL-10, TNF-α) in the VAT of pregnant women according to GWG and (2) explore whether VAT inflammation and GWG are associated with offspring anthropometric measures at birth.

## 2. Materials and Methods

Study population

This is a cross-sectional study conducted at the Hospital of Gynecology and Obstetrics 3, National Medical Center La Raza, and at the Hospital of Gynecology and Obstetrics 221, Instituto Mexicano del Seguro Social, and was approved by the Institutional Review Board (R-2018-785-026). All participants signed informed consent.

The study included 50 women aged 18 to 40 with singleton pregnancies and scheduled cesarean sections who delivered term infants. Indications for cesarean section were breech presentation and previous cesarean section. Exclusion criteria were smoking during pregnancy, pre-existing diabetes mellitus, major maternal comorbidities, and fetal malformations discovered at routine ultrasound examinations.

Maternal body mass index (BMI) was calculated from the weight measured at the first antenatal visit using a calibrated scale and the measured maternal height. BMI was categorized according to the WHO definitions: underweight < 18.5 kg/m^2^, target weight 18.5–24.9 kg/m^2^, overweight 25.0–29.9 kg/m^2^, and obesity ≥ 30 kg/m^2^. GWG was calculated as the difference between the weight measured at the first and last antenatal visit. GWG classification was determined using the IOM guidelines [7] and categorized as insufficient, adequate, or excessive GWG. Information on pregnancy was collected from hospital records.

Newborn anthropometric measurements (weight, length, foot length, head circumference, chest circumference, and abdominal circumference) were assessed at birth. The ponderal index was calculated as 100 × [birthweight (g)/length (cm^3^)].

Sample collection and biochemical analyses

Blood samples were collected in the morning on the day of the scheduled cesarean section after an overnight fast of 12 h. Glucose, total cholesterol, high-density lipoprotein (HDL) cholesterol, and triglyceride serum levels were determined using an ARCHITECT Plus c4000 Clinical Chemistry Analyzer (Abbot Diagnostics, Abbott Park, IL, USA). Low- density lipoprotein (LDL) cholesterol levels were estimated with the Friedewald formula. According to manufacturer’s guidelines, insulin levels were determined with a multiplex immunoassay using Magpix technology (Milliplex Map, Billerica, MA, USA). Homeostatic Model Assessment for Insulin Resistance (HOMA-IR) was estimated using the formula HOMA-IR = [fasting insulin concentration (µU/mL) × fasting glucose concentration (mmol/L)]/22.5 [15].

Gene expression analyses

Omental VAT fragments of about 5 cm^3^ of volume were obtained from all participants within ten minutes of delivery, and they were dissected into small fragments, placed in TRIzol^®^ Reagent (In-vitrogen^TM^, Carlsbad, CA, USA), and stored at −70 °C until RNA extraction. A Direct-zolTM RNA MiniPrep kit (Zymo Research Corp, Irvine, CA, USA) was used for total RNA extraction in accordance with the manufacturer’s protocol. RNA purity and quantity were measured using spectrophotometry (NanoDrop 2000, Thermo Fisher Scientific, Wilmington, DE, USA). cDNA was generated from RNA using a SuperScript^®^III First-Strand kit (Invitrogen^TM^, Carlsbad, CA, USA) following the manufacturers’ specifications. Subsequently, real-time PCR was performed using predesigned Taqman^®^ Gene Expression Assays and Taqman^®^ Universal PCR Master Mix (Applied Biosystems^TM^, Foster City, CA, USA). The 2^−ΔCt^ relative method quantification was used to determine the fold change in the mRNA expressions with the GAPDH transcript as endogenous control. All the primers and probes were acquired from Applied BiosystemsTM: TLR2 (Hs02621280_s1), TLR4 (Hs00152939_m1), NFκB (Hs00765730_m1), IKKβ (Hs01559460_m1), IL-1RA (Hs00893626_m1), IL-1β (Hs01555410_m1), IL-6 (Hs00985639_m1), IL-10 (Hs00961622_m1), TNF-α (Hs01113624_g1), and GAPDH (PN 4326317E).

Statistical analysis

Statistical analyses were performed using IBM SPSS Statistics 23.0 (IBM SPSS Inc., Chicago, IL, USA). The normality was tested with Shapiro–Wilk tests. Data are presented as median (25th and 75th percentiles). Comparisons between groups were analyzed using Kruskal–Wallis test followed by a post hoc test for multiple group comparisons. The relationship of VAT gene expression and offspring anthropometric measures was analyzed using the Spearman test. Multiple linear regression was applied to analyze the correlations of GWG (independent variable) and birth weight (dependent variable) after adjusting for maternal age, gestational age at delivery, pregestational maternal weight, parity, maternal metabolic factors (glucose, insulin, and lipid profile), and sex of the newborn. A two-tailed *p* value < 0.05 was considered statistically significant.

## 3. Results

Participant characteristics

The maternal characteristics are presented in Table 1. Most women were in their second or subsequent pregnancy; 57.5% had overweight or obesity at the onset of pregnancy, and 56.8% had adequate GWG. Fasting plasma glucose and insulin resistance were in the normal healthy range; however, women had a dyslipidemic profile (total cholesterol > 5.2 mmol/L and triglycerides > 1.7 mmol/L). These biochemical measures were no different among the GWG groups (Table 2).

Women with overweight/obesity at the onset of pregnancy had a higher proportion of excessive GWG than women with a target weight (24.1% vs. 17.6%, *p* = 0.009). None of the underweight women had excessive GWG; 75% gained below the recommended level. Moreover, 29% of target-weight women and 17.4% of overweight women had GWG below the guidelines.

Inflammatory gene expression and GWG

**Figure 1:**
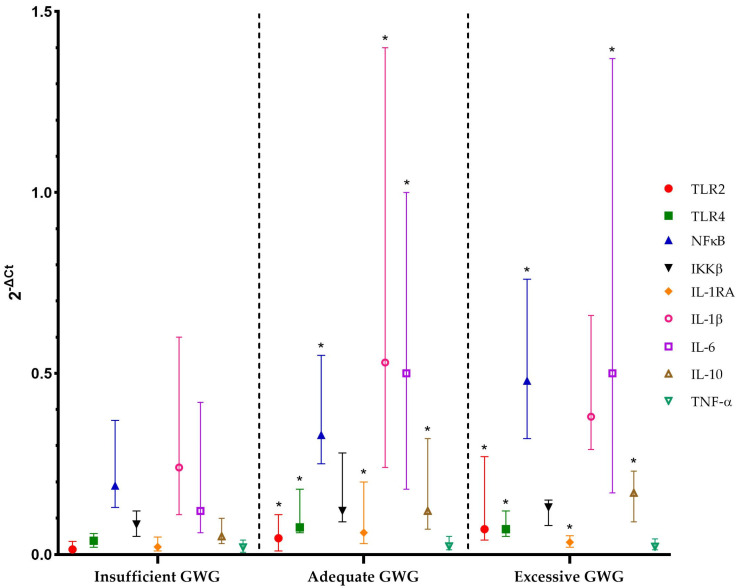
Inflammatory markers’ mRNA expression (Q1, median, and Q3 of 2^−ΔCt^) in VAT according to GWG. GWG: gestational weight gain. * *p* < 0.05 vs. insufficient GWG.

As shown in Figure 1, GWG was a relevant factor associated with the expression of genes involved in VAT inflammation. Women with excessive GWG had significantly higher TLR2, TLR4, IL-1RA, IL-6, IL-10, and NFκB expression than women with insufficient GWG. The GAPDH Ct values were not different among the groups [insufficient GWG: 23.8 (23.5–24.1); adequate GWG: 23.9 (23.3–24.3); excessive GWG: 23.6 (23.1–23.8); *p* = 0.72].

Also, women with adequate GWG had higher TLR2, TLR4, IL-1β, IL-1RA, IL-6, IL-10, and NFκB expression than women with insufficient GWG. No differences in gene expression were observed between women with adequate GWG and those with excessive GWG. Concerning overweight/obesity at the onset of pregnancy, no association was observed between maternal pregestational BMI and gene expression in VAT.

GWG and offspring anthropometric measures

Neonates from mothers with excessive GWG had greater birth weights and chest circumferences than those from mothers with insufficient GWG, and newborns from mothers with adequate GWG had higher head circumferences than those from mothers with insufficient GWG (Table 3). No differences in offspring anthropometric measures were observed between women with adequate GWG and those with excessive GWG.

GWG was positively correlated with birth weight (r = 0.288, *p* < 0.05), and this correlation still existed after adjusting for maternal age, gestational age at delivery, pregestational maternal weight, parity, maternal metabolic factors, and sex of the newborn (β = 0.4, *p* < 0.05).

Gene expression related to VAT inflammation was not associated with offspring anthropometric measures.

## 4. Discussion

A meta-analysis of more than 1.3 million pregnancies worldwide reported that only 28–32% of pregnant women have adequate GWG [2]. In Mexico, the prevalence of excessive GWG ranges from 38% to 43%, and this frequency is higher in women with overweight and women with obesity [16,17]. Our results showed that 56.8% of the participants had adequate GWG, 22.7% had insufficient GWG, and 20.5% had excessive GWG. This frequency is lower than that in previous studies, probably due to the age and the pregestational BMI range in our study (BMI range: 15.24–32.81 kg/m^2^). Additionally, we found that women with a higher pregestational BMI had higher GWG than those with a target weight. However, we did not observe significant differences in biochemical measures among the GWG groups. One explanation for this result could be the small sample size.

Maternal obesity is associated with impaired VAT function. We have previously reported that inflammatory genes in the VAT of women with obesity are upregulated compared with those in lean women late in pregnancy [18]. Inflammation during pregnancy has also been linked to GWG; recent studies have shown that the systemic inflammatory biomarkers C-reactive protein and interleukin 8 are positively correlated with GWG [19,20]. It has been suggested that inflammation stimulates the production of reactive oxidative species, which promote the expansion and terminal differentiation of adipocytes and sustained inflammation, leading to weight gain [21,22]. In the context of adipose tissue, TLR4 and TLR8 are involved in saturated fatty acid-mediated inflammation [23,24], IL-6 promotes macrophage infiltration [25], IL-1β is produced by activated macrophages [26], and NFκB is a transcription factor that triggers proinflammatory pathways in immune cells [27]. In turn, IL-10 is essential in resolving inflammation and subsequent tissue damage. IL-1RA inhibits the proinflammatory effects of IL-1, thus acting as an anti-inflammatory mediator [28]. We observed an up-regulation of most pro-inflammatory and of all anti-inflammatory genes in women with adequate or excessive GWG compared with the group with insufficient GWG. The down-regulation of these genes in insufficient GWG may reflect an energetic maladaptation of VAT that could partly explain the high risk of small-for-gestational-age infants [2,29]. Moreover, there may have been a difference in the pattern of adipose distribution between those women with insufficient GWG and those with adequate and excessive GWG; lean women gain more subcutaneous adipose tissue, and women with overweight and women with obesity have a greater proportion of VAT during pregnancy [30].

Interestingly, it has been suggested that maternal inflammation should contribute to the fetal programming of obesity through changes in organs that release pro-inflammatory cytokines as AT and the placenta [31]. Notably, in our study, the increased expression of the inflammatory cytokines in VAT was not associated with anthropometric neonatal measures. However, there is evidence that the placenta substantially contributes to maternal cytokine concentrations and nutrient transport to the fetus [32].

Several studies have shown that excessive GWG and pregestational BMI are associated with offspring birth weight and adiposity [2,13]. However, in our study, we did not observe an association between BMI and newborn weight. Only GWG was found to be independently and positively associated with newborn weight. Excessive GWG is accompanied by increased fat mass associated with birthweight because of the increased availability of plasma fatty acids for placental transport [33].

The present study is strengthened by the adjustment for several major elements that may confound the relationship between GWG and neonatal anthropometric measures. However, our study has some limitations. We had a modest sample size, which may limit the study’s power to identify GWG-related metabolic profiles. Additionally, we were unable to obtain information regarding dietary patterns, lifestyle behaviors, and socioeconomic status during pregnancy. Newborn and maternal fat mass were not examined. At the same time, we investigated the overall GWG at the end of pregnancy instead of weight gain during each trimester. It has been suggested that GWG during the first and early second trimesters is related to somatic growth [34]. Finally, although our study demonstrates associations, we cannot determine causation.

## 5. Conclusions

The expression of most genes associated with inflammatory pathways was higher in excessive and adequate GWG than in pregnant women with insufficient GWG. However, VAT inflammation was not related to offspring anthropometric measures. Further longitudinal studies on bigger samples are required to evaluate the role of VAT inflammation on maternal and neonatal outcomes.

Additionally, GWG was found to be positively associated with newborn weight. This suggests that interventions, information, and counseling to optimize GWG should be evaluated for their ability to reduce excessive fetal growth.

## Figures and Tables

**Table 1:** Anthropometric and laboratory characteristics of the study group.

Characteristics	*n* = 50
Maternal age at delivery (years)	30.0 (22.0–33.3)
Previous pregnancies (%)	
None	31.9
At least one	68.1
Pre-pregnancy BMI *n* (%)	
<18 Underweight	4 (8.5)
18.0–24.9 Target weight	17 (34.0)
25.0–29.9 Overweight	23 (44.7)
>30 Obesity	6 (12.8)
Gestational weight gain *n* (%)	
Insufficient	12 (22.7)
Adequate	28 (56.8)
Excessive	10 (20.5)
Gestational age (weeks)	39.0 (38.0–40.0)
Fasting glucose (mmol/L)	4.2 (3.8–5.1)
Fasting insulin (pmol/L)	51.8 (30.1–73.4)
HOMA-IR	1.3 (0.82–2.0)
Total cholesterol (mmol/L)	6.1 (5.2–6.7)
HDL cholesterol (mmol/L)	2.7 (2.3–3.1)
LDL cholesterol (mmol/L)	1.9 (1.2–2.3)
Triglycerides (mmol/L)	3.0 (2.5–3.6)

Data are presented as counts and percentages as well as medians (interquartile range). BMI: body mass index, HOMA-IR: homeostasis model assessment-insulin resistance, HDL: high-density lipoprotein cholesterol: LDL low-density lipoprotein cholesterol.

**Table 2:** Biochemical parameters according to GWG.

	Insufficient GWG	Adequate GWG	Excessive GWG
Fasting glucose (mmol/L)	4.3 (3.6–6.2)	4.2 (3.8–4.5)	4.8 (3.7–5.4)
Fasting insulin (pmol/L)	55.9 (42.7–75.2)	51.1 (27.4–79.2)	50.3 (36.4–103.9)
HOMA-IR	1.3 (0.9–2.2)	1.3 (0.7–2.2)	1.4 (0.9–2.1)
Total cholesterol (mmol/L)	6.6 (5.6–7.5)	5.6 (5.0–6.5)	6.2 (5.4–6.8)
HDL cholesterol (mmol/L)	2.8 (2.4–3.4)	2.5 (2.3–3.2)	2.5 (2.2–3.0)
LDL cholesterol (mmol/L)	2.2 (1.3–3.7)	1.7 (1.1–2.1)	1.9 (1.0–3.1)
Triglycerides (mmol/L)	3.1 (2.6–3.7)	2.9 (2.4–3.6)	3.3 (2.8–4.0)

Data are presented as medians (interquartile range). HOMA-IR: homeostasis model assessment-insulin resistance, HDL: high-density lipoprotein cholesterol, LDL: low-density lipoprotein cholesterol.

**Table 3:** Offspring anthropometric measures according to GWG.

	Insufficient GWG	Adequate GWG	Excessive GWG
Gestational age (weeks)	39 (37.3–39.8)	38.9 (38.0–39.5)	39 (38.5–40.3)
Sex *n* (%)			
Female	9 (75.0)	9 (34.6)	6 (66.7)
Male	3 (25.0)	17 (65.4)	3 (33.3)
Birth weight (g)	2850 (2352.5–3100)	3025 (2787.5–3202.5)	3150 (3020–3395) *
Birth length (cm)	49 (48–50)	49 (47–50)	48 (47.25–51.25)
Ponderal index (g/cm^3^)	2.47 (2.15–2.8)	2.63 (2.4–2.8)	2.67 (2.46–2.85)
Neonatal foot length (cm)	7.5 (7–8)	8 (7–8)	8 (7–8)
Neonatal head circumference (cm)	33 (32–34)	34 (33–35) *	34 (32.5–35.25)
Neonatal chest circumference (cm)	31 (29–32.75)	32 (31–33)	34.5 (32.0–35.0) *
Neonatal abdominal circumference (cm)	29 (28–30.75)	30.5 (29.5–32.25)	30.0 (29.0–33.5)

Data are presented as medians (interquartile range). * *p* < 0.05 vs. insufficient GWG.

## Data Availability

The data used to support the findings of this study are available from the corresponding author by request.
